# Tunable two-dimensional assembly of colloidal particles in rotating electric fields

**DOI:** 10.1038/s41598-017-14001-y

**Published:** 2017-10-23

**Authors:** Egor V. Yakovlev, Kirill A. Komarov, Kirill I. Zaytsev, Nikita P. Kryuchkov, Kirill I. Koshelev, Arsen K. Zotov, Dmitry A. Shelestov, Victor L. Tolstoguzov, Vladimir N. Kurlov, Alexei V. Ivlev, Stanislav O. Yurchenko

**Affiliations:** 1 Bauman Moscow State Technical University, 2nd Baumanskaya street 5, 105005 Moscow, Russia; 2 Institute of Solid State Physics of Russian Academy of Sciences, Academician Osipyan street 2, 142432 Chernogolovka, Russia; 3 Max-Planck-Institut für extraterrestrische Physik, Giessenbachstrasse 1, 85748 Garching, Germany

## Abstract

Tunable interparticle interactions in colloidal suspensions are of great interest because of their fundamental and practical significance. In this paper we present a new experimental setup for self-assembly of colloidal particles in two-dimensional systems, where the interactions are controlled by external rotating electric fields. The maximal magnitude of the field in a suspension is 25 V/mm, the field homogeneity is better than 1% over the horizontal distance of 250 *μ*m, and the rotation frequency is in the range of 40 Hz to 30 kHz. Based on numerical electrostatic calculations for the developed setup with eight planar electrodes, we found optimal experimental conditions and performed demonstration experiments with a suspension of 2.12 *μ*m silica particles in water. Thanks to its technological flexibility, the setup is well suited for particle-resolved studies of fundamental generic phenomena occurring in classical liquids and solids, and therefore it should be of interest for a broad community of soft matter, photonics, and material science.

## Introduction

Self-assembly of colloidal particles with sizes of $\sim 10\,{\rm{nm}}$ to $\sim 10\,{\rm{\mu }}m$ is a very common phenomenon in nature. Hierarchical (multiscale) order occurring through self-assembly is governed by local interactions between particles, which can be used in prospective materials, devices, and technologies. The assembled materials have emergent properties, making them appealing for photonics, electronics, and sensing technologies^1^.

Self-assembly of particles can be driven by different mechanisms, such as their interaction energy, entropy, and external fields^2^. The process strongly depends both on specific properties of individual particles and their surrounding, and can be due to gravitational field^3,4^, confinement at structured substrates^5,6^ and interfaces^7–10^, chemical heterogeneities in solvents^2,11^, electrophoresis^12–15^, dielectrophoresis^16–18^, depletion forces^19^, optical fields^20,21^, magnetic fields^22–29^, and alternating anisotropic and rotating electric fields^6,30–40^. Self-assembly occurs relatively easy at nano- and micro-scales^2^. However, various technological applications need controllable self-assembly at scales over mm. The use of alternating electric fields provides a number of advantages for this purpose. For instance, electric fields can be created and controlled accurately at large spatial scales by a system of compact electrodes. Contrary to magnetically-driven self-assembly, where specific magnetic properties of colloids and solvent are required, the only necessary condition for electrically-induced interparticle interactions is a contrast between complex dielectric permittivities of the particles and solvent.

**Figure 1:**
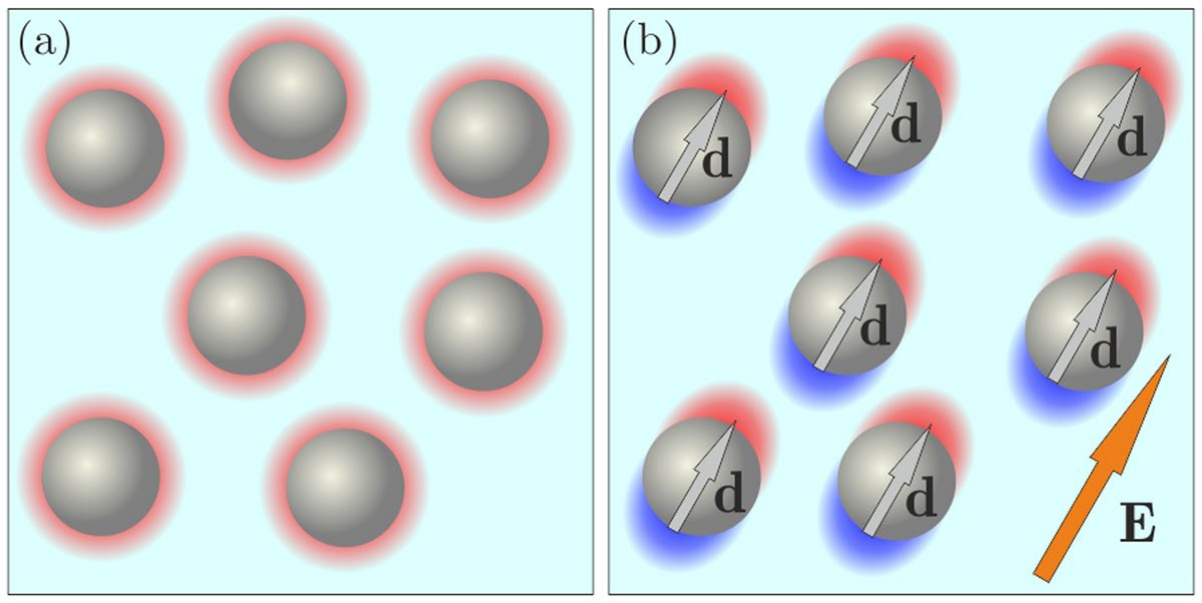
Schematic illustration of interparticle interactions induced by an external electric field. (**a**) In the absence of the field, each particle (typically charged negatively) is surrounded by a spherical cloud of screening ions (predominantly positive cations, shown in red). (**b**) External field ${\bf{E}}$ polarizes each particle and also the surrounding ion cloud (as indicated by the red-blue shading displaced along the field direction).

The mechanism of the field-induced attraction between particles can be generally understood as follows: External field ${\bf{E}}$ polarizes colloidal particles (electric field also induces currents of cations and anions in the solvent, thus polarizing the surrounding ion clouds), as illustrated in Fig. 1. The energy of dipole-dipole interaction between two identical particles located at the relative distance ${\bf{r}}$ is ${\varphi }({\bf{r}},\theta )={d}^{2}(1-3{\cos }^{2}\theta )\mathrm{/4}\pi {\varepsilon }_{0}{\varepsilon }_{S}{r}^{3}$, where ${\bf{d}}\propto {\bf{E}}$ is the induced dipole moment of a particle, $\theta$ is the angle between ${\bf{E}}$ and ${\bf{r}},\,{\varepsilon }_{0}$, is the vacuum permittivity, and ${\varepsilon }_{S}$ is the dielectric permittivity of the solvent. If the field rotates in the particle plane with a sufficiently high frequency (larger than the inverse relevant diffusion timescale of a particle), one can average ${\varphi }({\bf{r}},\theta )$ over $\theta$ and obtain the effective interaction energy. This leads to isotropic tunable attraction^31^, ${\varphi }(r)\propto -{E}^{2}/{r}^{3}$. Due to the long-range nature of dipolar interactions, the particle self-assembly can be realized over a large area.

Apart from their practical applications, colloidal suspensions are known to be used as model systems for particle-resolved studies of molecular systems^41–46^. Colloidal suspensions with tunable attractive interactions are particularly well suited for investigating generic phenomena in liquids and solids, e.g., for studying the role of attraction in melting and crystallization, binodal decay, dynamics of dislocations, solid-solid phase transitions, nucleation, etc.^41,45^. However, an external rotating field controlling the long-range attraction should be very homogeneous across the suspension, to ensure quasi-identical interactions in the whole system.

Here, we present a new experimental setup for tunable self-assembly of particles in horizontal two-dimensional (2D) colloidal suspensions. The interparticle attraction is driven by a horizontally rotating electric field, with the maximal magnitude in a suspension of 25 V/mm, homogeneity better than 1% over the horizontal distance of 250 *μ*m, and the rotation frequency in the range of 40 Hz to 30 kHz. Using numerical calculations, we carefully investigate the spatial distribution of the field generated by eight planar electrodes (located at the same height). The distribution changes qualitatively with the vertical distance from the electrode plane, revealing a remarkable horizontal homogeneity at a certain height and thus providing optimal experimental conditions. To demonstrate this regime, we perform experiments on self-assembly of 2.12 *μ*m silica particles in water. Our simulations and experiments show that the proposed setup has excellent characteristics for prospective studies–in particular, a high homogeneity and magnitude of the field, as well as tunability of the frequency and form of the rotating field. The setup has been primarily developed for controlled particle-resolved studies of large 2D colloidal systems. However, based on the achieved characteristics, we suggest that it can also be utilized for applied research in soft matter, photonics, material science, chemical physics, microfluidics, and biological systems.

## Results

**Figure 2:**
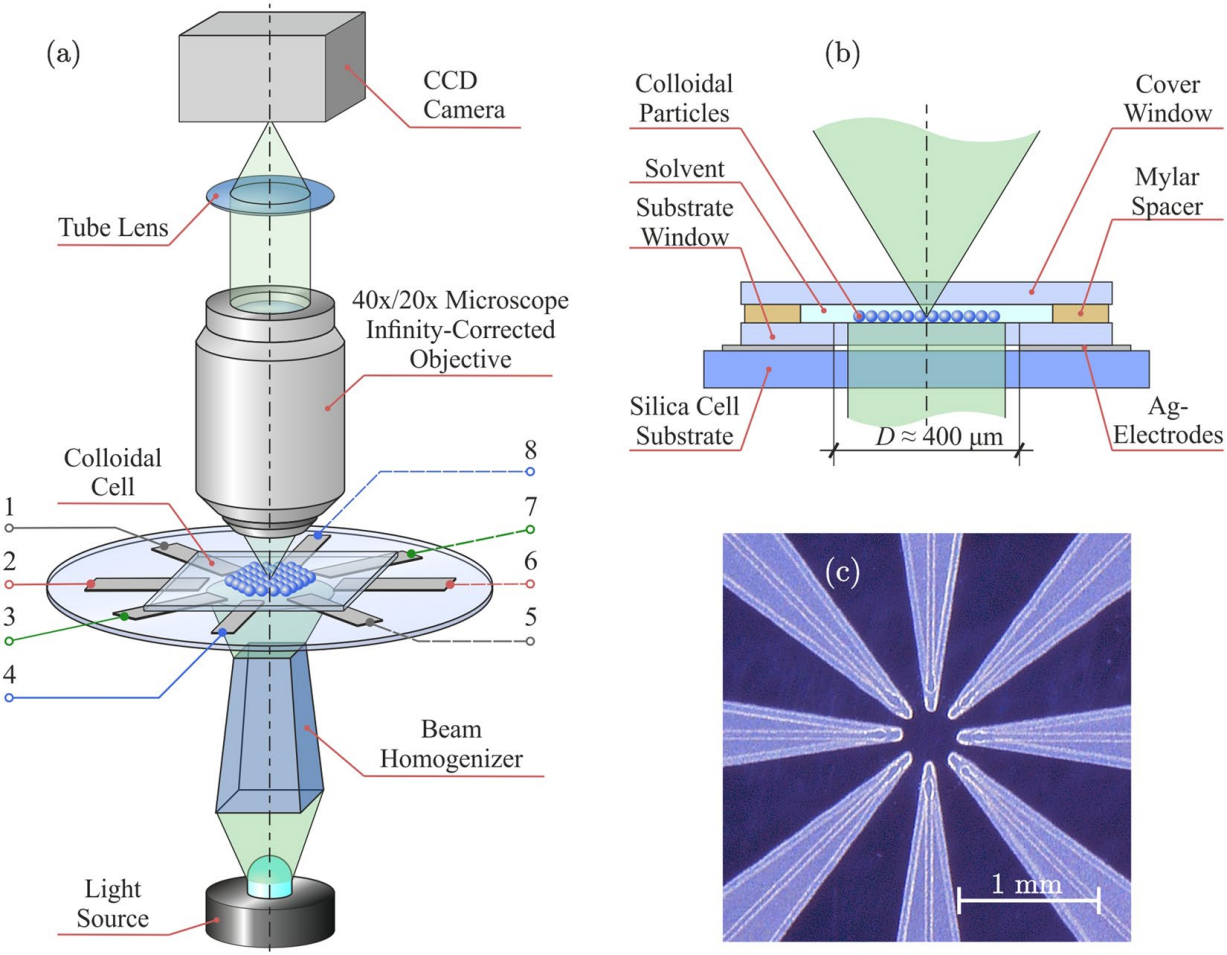
Schematic representation of the experimental setup for tunable self-assembly of two-dimensional colloidal suspensions in rotating electric fields: Panel (a) shows an overall scheme of the setup with details presented in (**b**), panel (c) is a photo of eight-electrodes cell (8EC). Potentials ${{\phi }}_{k}$ at electrodes 1–8 in panel (a) are controlled by a module of electric signal generation (see Methods for technical details).

Figure 2 presents the developed experimental setup for tunable self-assembly of colloidal particles in external rotating electric fields. The setup includes an optical module and colloidal cell shown in the figure, as well as a module of electrical signal generation described in Methods. Through the optically transparent cell, the colloidal suspension is illuminated using light source and beam homogenizer. The video-imaging is provided by CCD Camera (8051C-GE, Thorlabs) using a $40{\rm{x}}$ (RMS40X-PF, Olympus) or $20{\rm{x}}$ (RMS20X-PF, Olympus) microscope infinity-corrected objective and a tube lens. A translation stage enables accurate positioning of the cell both in the vertical and lateral directions.

The colloidal cell is sketched in Fig. 2(b). The work space with a colloidal suspension is bounded by substrate and cover windows (with sizes of $20\times 20\times 0.15\,{{\rm{mm}}}^{3}$), which are separated by $10$-*μ*m-Mylar polymer spacer placed at the periphery of the windows. A silica substrate, at which the $5$-*μ*m-thick silver electrodes are deposited by aerosol sputtering method, is attached to the substrate window from below. The electrodes have a form of arrows with angle of 15° and radius of curvature of 25 *μ*m in the vertex, converging radially to the center of the cell, as shown in Fig. 2(c). The distance between the vertexes of opposite electrodes is $D=400\,{\rm{\mu }}m$.

The vertical distance from the electrode plane to the plane of particle self-assembly can be easily controlled by varying the thickness of the substrate window. Four pairs of independent signals with frequencies and magnitudes of $\mathrm{(0.04}\ldots \mathrm{30)}\times {10}^{3}\,{\rm{Hz}}$ and $2.5\times \mathrm{(1}\ldots {10}^{3})\,{\rm{V}}$, respectively, can be applied to the electrodes using the module of electric signal generation (see Methods). With the presented experimental setup one can flexibly tune the magnitude, homogeneity, rotation frequency, and hodograph of the electric field in a suspension.

The spatial distribution of the electric field generated in the cell plays the crucial role in self-assembly of particles. The main advantage of the proposed eight-electrode cell (8EC) is the ability to produce homogeneous electric fields. To study the field distribution, we performed simulations using boundary element method (see Methods for details). Since the charge distributions at biased neighboring electrodes are mutually affected, the electrostatic boundary problem has to be solved for a given electrode configuration. As a basic example, we considered a circular distribution of potentials applied to the electrodes,

1 $${{\phi }}_{k}(t)=\frac{U}{2}\,\cos \,[(k-\mathrm{1)}\frac{\pi }{4}+\alpha (t)],$$

where $U$ is the voltage amplitude, $k$ is the electrode number (see Fig. 2(a)), and $\alpha (t)$ is a time-dependent phase shift. For uniformly rotating fields $\alpha (t)=2\pi \nu t$, where $\nu$ is the rotation frequency.

**Figure 3:**
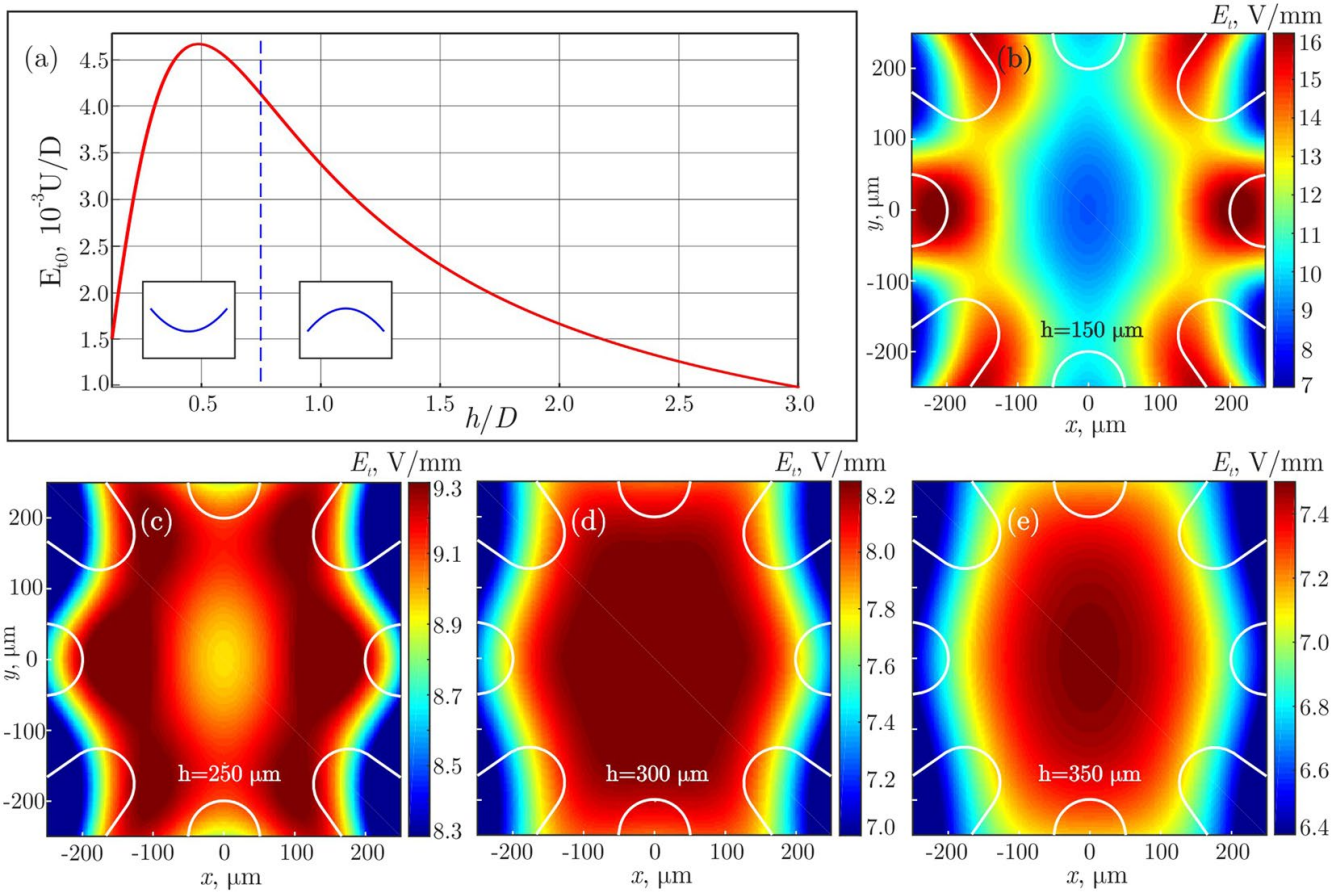
Numerical simulation of the electric field in 8EC: Panel (a) shows the magnitude of the tangential electric field in the cell center, ${E}_{t0}$, versus the normalized thickness of substrate window, $h/D$. The vertical dashed line at $h/D\simeq 0.75$ marks the critical (optimum) height where the field configuration in the center changes its curvature. Panels (b)–(e) demonstrate the horizontal distributions of ${E}_{t0}$ for different $h$. The white solid lines indicate contours of the electrodes from the top view. Calculations are performed for $D=400\,{\mu }{\rm{m}}$ and $U/D=2\,{\rm{V}}/{\mu }{\rm{m}}$, the voltage distribution ${\phi }_{k}$ at the electrodes is given by Eq. (1) for $\alpha =0$. The optimal field configuration is obtained for $h=300\,{\mu }{\rm{m}}$ ($h/D=3/4$): The field homogeneity is better than 1% over the horizontal distance of $\simeq 250\,{\mu }{\rm{m}}$, as one can see from panel (d).

**Figure 4:**
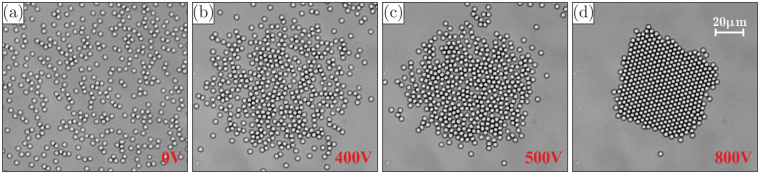
A demonstration experiment with a self-assembly of colloidal particles in external rotating electric fields: Panels (a)–(d) depict about 450 silica particles of $2.12\,{\mu }{\rm{m}}$ diameter, undergoing a transition from a (dilute) fluid state to a crystalline state upon a gradual increase of the field ($U=\mathrm{0,}\,\mathrm{0.4,}\,\mathrm{0.5,}\,0.8$ kV, respectively). In the shown example the field rotated with the frequency of $\nu =30\,{\rm{kHz}}$.

Figure 3 shows the calculated distribution of electric field in 8EC. At a certain height, presented in Fig. 3(d), we observe a remarkable homogeneity of the field over a large horizontal area. To check the predictions, we carried out a demonstration experiment with suspensions of silica particles in water (see details in Methods). A typical cluster observed in the experiment is illustrated in Fig. 4.

## Discussion

Figure 3 demonstrates results of the calculations of the horizontal electric field, representing our typical experimental conditions. We found that the vertical component of electric field is much smaller than the horizontal one, because of two reasons: (i) the electric field is horizontal in the center due to symmetry, and (ii) a large dielectric contrast between the substrate window and aqua-based solvent leads to a significant reduction of the vertical field component in the solvent. For instance, the absolute value of the vertical-to-horizontal field ratio is less than ${10}^{-2}$ for substrate window thickness $h=150\,{\mu }{\rm{m}}$.

Figure 3(a) shows the dependence of the tangential (in-plane) magnitude of electric field ${E}_{t0}$ in the center of self-assembly plane on thickness $h$ of the substrate window. One can see that the used planar electrodes yield a non-monotonous dependence, reaching a maximum at $h/D\simeq 0.5$. Figures 3(b)–(e) present distributions of the tangential electric field in the plane of self-assembly at $h=\mathrm{150,}\,\mathrm{250,}\,300\,{\rm{and}}\,350\,{\mu }{\rm{m}}$, corresponding, respectively, to $h/\,D=\mathrm{3/8},\,\mathrm{5/8},\,\mathrm{3/4}\,{\rm{and}}\,\mathrm{7/8}$, in Fig. 3(a). At small $h$, the tangential field has a local minimum in the center, as shown in Fig. 3(b), while for thick substrate windows, Fig. 3(e), the field attains a maximum in the center. Thus, varying $h$ provides a simple way for optimization of experimental conditions.

The *most homogeneous* distribution of the electric field, presented in Fig. 3(d), is obtained at $h/D\simeq \mathrm{3/4}$. Near this value the surface of the constant field magnitude changes its curvature, as shown schematically in Fig. 3(a). We found that in this case ${E}_{t0}$ changes in the center by less than 1% over the horizontal distance of $\simeq 250\,{\mu }{\rm{m}}$ (for $D=400{\mu }{\rm{m}}$).

Although the maximum field is reached at $h/D\simeq 1/2$, the cell configuration with $h/D\simeq 3/4$ provides the most promising conditions for experimental studies: the largest area of a homogeneous electric field in the plane of self-assembly combined with a reasonably high magnitude of the field. Indeed, according to Fig. 3(a), the maximum field $\simeq 4.6\times {10}^{-3}U/D$ corresponds to $\simeq 28$ V/mm for $D=400{\mu }{\rm{m}}$ and $U=2.5$ kV. For the optimal configuration shown in Fig. 3(d), the magnitude of the field is only slightly smaller, ${E}_{t0}\simeq 4.1\times {10}^{-3}U/D$, corresponding to $\simeq 25$ V/mm.

During the rotation, the field varies periodically between two configurations corresponding to $\alpha =0$ and $\alpha =\pi \mathrm{/8}$ in Eq. (1). Calculations performed for $\alpha =\pi \mathrm{/8}$ reveal practically the same field configuration as in Fig. 3, just rotated by $\alpha =\pi \mathrm{/8}$.

In our demonstration experiment illustrated in Fig. 4, we observed in total about 10 very isotropic clusters, as follows from the analysis of the angular distributions of distances between the neighbor particles. Results of self-assembly at different values of $U$ are presented in Figs. 4(a)–(d) for a typical equilibrium cluster (the system was equilibrated over 3–5 min after each voltage change). Here, we provide snapshots of the center of a self-assembly plane, with the field of view of $130\times 130{\mu }{{\rm{m}}}^{2}$. Figures 4(a)–(d) demonstrate that, by applying in-plane rotating electric field of increasing magnitude, particles can be assembled in a complex two-dimensional liquid cluster which crystallizes when $U$ is sufficiently large. The spatial distribution of electric field in the experiment corresponds to that shown in Fig. 3(d). The crystal melts after the field is turned off, then the whole process of self-assembly can be repeated. Note that the field-controlled reversibility distinguishes this mechanism of self-assembly from other mechanisms, typically driven by chemical reactions.

**Figure 5:**
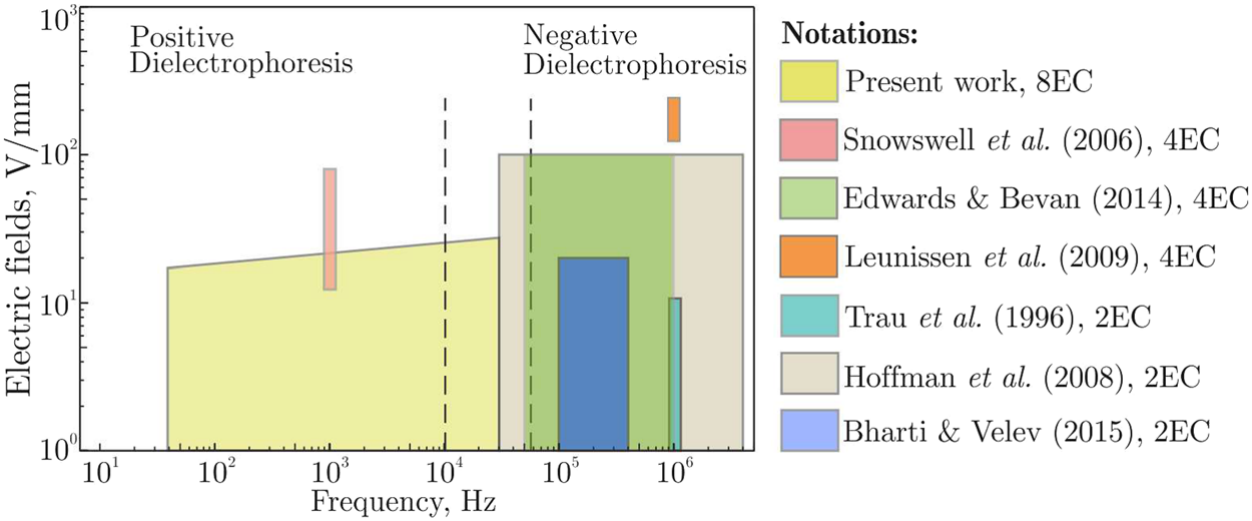
Comparison of 8EC with other setups in terms of the field frequency and magnitude.

Let us compare the main characteristics of our experimental setup with those reported in other papers. In particular, we consider the magnitude of electric field, rotation frequency, and field homogeneity achieved in the plane of self-assembly. Figure 5 presents the operation regimes of the setups in terms of the field magnitude and frequency. We have chosen these characteristics since the sign of dielectrophoresis may change with the frequency^17,18^, while the field strength determines the magnitude of induced interparticle interactions. The maximal voltage generated by amplifiers used in our setup slightly increases with the frequency (see Methods for details), as indicated in Fig. 5. Using available data of refs.^12,15,30,31,34,40,47^, the electric field in solvent for other experimental setups is estimated as $U/D$, where $U$ is a voltage amplitude at electrodes and $D$ is the distance between them. In Table 1 we summarize relevant parameters, such as the cell size, characteristic size of area with a homogeneous electric field, range of frequencies, and maximum magnitude of the field in solvent.

Frequencies in Fig. 5 are divided into the low- and high-frequency regimes, corresponding to the essentially positive and negative dielectrophoresis emerging at $\nu \mathop{ < }\limits_{ \tilde {}}10\,{\rm{kHz}}$ and $\nu \mathop{ > }\limits_{ \tilde {}}56{\rm{kHz}}$ ^17,18^, respectively. One can see that in the low-frequency regime our setup enables particle manipulation via a positive dielectrophoresis (where particles move toward the field maximum). In the frequency range between $\simeq 10$ kHz and $\simeq 56$ kHz the dielectrophoresis is practically negligible, providing the best conditions for self-assembly due to the field-induced interactions. For instance, the demonstration experiment in Fig. 4, corresponding to the optimal thickness $h/D=\mathrm{3/4}$, was performed at frequency $\nu =30\,{\rm{kHz}}$.

According to our simulations, the number of electrodes and their geometry strongly affect the field configuration: For four-electrode cells, the horizontal field varies by less than 1% is in the central area of $\simeq 60\,{\mu }{\rm{m}}$ size (compared to $\simeq 250\,{\mu }{\rm{m}}$ in 8EC with otherwise the same conditions). Hence, the proposed setup provides much more homogeneous electric fields and therefore enables self-assembly over much larger area than in setups reported in refs.^6,30,31,34,43.^ Furthermore, 8EC is more flexible technologically, since it can be restricted to operation in regimes with four and two electrodes. Therefore, in Fig. 5 and in Table 1 we also present the reference data for 2EC, although the assembled structures were essentially anisotropic in these cases. Note that Leunissen *et al*.^40^ used four-electrode capillary cell, but the particles were assembled in the vertical plane.

**Table 1:** Main parameters of the present setup and setups used in previous studies.

Paper	${N}_{EC}^{\ast }$	Cell size, $L$	${L}_{1}^{\ast \ast }$	Frequencies, Hz	Maximal field, $V/\text{mm}$
Present work	8	$400\,\mu m$	$250\,{\mu }{\rm{m}}$	$\mathrm{(0.04}\ldots \mathrm{30)}\times {10}^{3}$	$25$
Snowswell *et al*. (2006)^30,31^	4	$650\,\mu m$	$\sim 90\,{\mu }{\rm{m}}$	${10}^{3}$	$80$
Edwards & Bevan (2014)^34^	4	$100\,\mu m$	$\sim 15\,\mu m$	$\mathrm{(0.5}\ldots \mathrm{10)}\times {10}^{5}$	$100$
Leunissen *et al*. (2009)^40^	4	$400\,\mu m$	—	${10}^{6}$	$600$
Trau *et al*. (1996)^15^	2	$200\,\mu m$	—	${10}^{6}$	$10$
Hoffman *et al*. (2008)^12^	2	$50\,\mu m$	—	$\mathrm{(0.2}\ldots \mathrm{40)}\times {10}^{5}$	$100$
Bharti & Velev (2015)^47^	2	$2000\,\mu m$	—	$500\ldots {10}^{6}$	$20$

*${N}_{EC}$ is the number of electrodes in the setup.

**${L}_{1}$ is the characteristic size of area within the cell, where the field homogeneity is better than 1%.

From Fig. 5 one can see that the magnitude of electric field in the developed setup is smaller than that in refs.^30,34^. This is because we used insulated electrodes, while in the other setups the electrodes were in direct contact with solvent. We point out that insulated electrodes prevent electrolysis, discharge in solvent^30^, and can be used with biological systems. Generation of strong fields is particularly important for self-assembly of small (nano) particles and particles with a small dielectric contrast (with respect to solvent), since the strength of electrically-induced interactions drops rapidly with decrease of these parameters^2^. However, our setup provides good conditions for self-assembly of even sub-micrometer particles in crystalline clusters: For instance, clusters in a suspension of $0.95$-${\mu }{\rm{m}}$ particles can be crystallized in the rotating field of ${E}_{t0}\simeq 15V/\text{mm}$ ^30,31^, which is well below the maximum value of $\simeq 25V/\text{mm}$ for our setup (in the optimal configuration shown in Fig. 3(d)). For $2.12$-${\mu }{\rm{m}}$ particles, crystallization occurs at ${E}_{t0}\simeq 6.2V/\text{mm}$. In addition, 8EC allows us to generate rotating fields with a complex (angularly-modulated) hodograph, to induce anisotropic interparticle interactions. This provides additional opportunities for tunable self-assembly of structures with complicated geometries.

The cluster shown in Fig. 4 contains more than 450 particles, which is almost twice as much as reported in refs.^12,15,31,34,40,47^. The colloidal crystals can be used, e.g., to grow photonic crystals, as structured substrates for sensors and light conversion, as matrices for optical composites, and for spectroscopy using optical field localization^48–51^. The same principle of field-induced self-assembly can be applied to bubbles stabilized by ions in aqua solutions of electrolytes^52–55^, or generated by intensive electric fields^56,57^. This mechanism may be utilized for novel technologies of deep degassing of aqua solutions^58^. However, such studies are beyond the scope of the present paper and should be considered in future works.

We hope that the presented experimental setup can be employed for fundamental and applied research in different fields, where tunable interactions and self-assembly in complex media are important. Apart from technological applications of 2D colloidal clusters, the setup is well suited for fundamental particle-resolved studies of collective phenomena in many-body systems. The ability to form large homogeneous systems of particles is crucial for experiments aiming to better understand the role of interparticle attraction in elementary processes governing nucleation, melting, crystallization, and dislocation dynamics.

## Methods

### Module of electric signal generation

**Figure 6:**
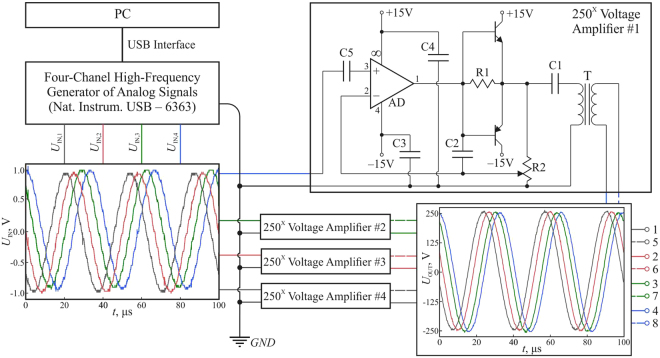
The principal scheme of electric signal generation.

The principal scheme of the module of electric signal generation is shown in Fig. 6. The module consists of a personal computer (PC) with a specially developed software, digital-to-analog converter (DAC), and 4-channel electrical voltage $250{\rm{x}}$ amplifier. The software allows us to generate predetermined profiles of the voltage signals at the electrodes. In addition, the frequency and magnitude of DAC signals can be varied in the ranges $0.04\ldots 30\,{\rm{kHz}}$ and $0.1\ldots 10\,{\rm{V}}$, respectively. Magnitude of the signal for each channel is amplified by a $250{\rm{x}}$ voltage amplifier. Each channel of amplifier consists of operational amplifier AD826 (Analog Devices, USA), current buffer based on complementary transistors BD139 (npn-type, ST Microelectronics, Switzerland), BD140 (pnp-type, ST Microelectronics, Switzerland), and setup transformer. Operational amplifier, working in non-inverting mode, and current buffer have the joint negative feedback, which essentially linearizes the system. Crossover distortion is prevented due to the feedback, which encloses transistors and operational amplifier. Resistor R1 (50 Om) is supplied for the best result of getting rid of the distortion. The amplification coefficient can be tuned by the potentiometer R2 ($10\,{\rm{k}}{\rm{\Omega }}$). The ceramic capacitor C2 ($68\,{\rm{pF}}$) prevents a high-frequency self-excitation of the operational amplifier. The voltage amplification occurs in transformer T (Iron-core plate-type power transformer TP-114–1, produced by Scientific and industrial company “Complex”; input/output voltage 220 V/6 V, nominal load current 2.1 A), which does not pass the high-frequency harmonics, because at 30 kHz (our main operating frequency) the gain reaches its maximum and then drops at higher frequencies, as shown in Fig. 5. The capacitor C1 plays a role of a separating capacitor, which blocks an undesirable constant component of the signal and prevents an overload of the transistors. The capacitor C5 is also a separating one, in the amplifier input. The values of C1 and C5 (in our case, ${\rm{C1}}={\rm{C5}}=10\,{\mu }{\rm{F}}$) were chosen from the condition that their reactive resistance at low frequencies does not affect the propagation of alternating signal. The ceramic capacitors C3 and C4 play a role of source filters and have a capacity of $0.1\,{\mu }{\rm{F}}$. The maximal voltage amplification of 250x is reached at frequency $30\,{\rm{kHz}}$, providing the maximum voltage difference of 2.5 kV at opposite electrodes. The input and output signals have the same form (when superimposed on the oscilloscope screen and scaled linearly to magnitude), the total harmonic distortion is less than 3%.

### Electrostatic fields in a colloidal cell

The complex dielectric permittivity of solvent can be written as^59^ $\varepsilon ={\varepsilon }_{W}-i\sigma \mathrm{/2}\pi {\varepsilon }_{0}\nu$, where ${\varepsilon }_{0}$ is the vacuum permittivity and ${\varepsilon }_{W}$ is a real part of the dielectric permittivity of water. The conductivity of deionized water (used in our experiment) is $\sigma \simeq 5\times {10}^{-5}\,\text{Sm}/\text{cm}$. At $\nu \simeq 3\times {10}^{4}\,{\rm{Hz}}$ (experimental operation frequency), $\sigma \mathrm{/2}\pi \nu {\varepsilon }_{0}{\varepsilon }_{W}\simeq 4\times {10}^{-2}\ll 1$. Therefore, our solvent can be considered as an insulator, and the electric fields in the cell can be calculated in the quasistatic limit.

**Figure 7:**
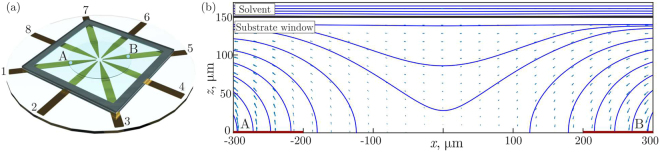
Example of electrostatic calculations: Panel (a) depicts a 3D model of 8EC, potential ${{\phi }}_{k}$ at electrode $k$ is given by Eq. (1). Panel (b) shows the field distribution in the vertical plane (for $h=150\,{\mu }{\rm{m}}$ and $\alpha =0$), through points A and B indicated in panel (a). Planar electrodes are marked at $z=0$ by the thick red solid lines, the interface between solvent and substrate window is the black solid line at $z=h$. The blue arrows represent the electric field, with the blue solid lines being the force lines.

Figure 7(a) shows a 3D model of the cell used for the calculations. Since the planar electrodes are very thin ($5{\mu }{\rm{m}}$) in comparison to the substrate window thickness $h$ and diameter $D$, one can solve the electrostatic problem assuming that the electrodes are located at $z=0$.

The electrostatic potential ${\phi }({\bf{r}})$ is given by the solution of the Laplace equation

2 $${\nabla }^{2}{\phi }({\bf{r}})=\mathrm{0,}$$

with the boundary condition for the potential in the plane of electrodes and at large distances:

3 $${\phi }({\bf{r}}{)|}_{z=0}=\{\begin{array}{cc}{\phi }_{k}, & {\rm{at}}\,{\rm{electrode}}\,k;\\ \mathrm{0,} & {\rm{otherwise}},\end{array}$$

4 $${\phi }({\bf{r}}{)|}_{z\to \pm \infty }\to 0.$$

The standard boundary conditions at the interface between dielectric areas 1 and 2 are^59^

5 $${{\phi }}_{1}={{\phi }}_{2},\quad {\varepsilon }_{1}{E}_{n1}={\varepsilon }_{2}{E}_{n2},\quad {E}_{t1}={E}_{t2},$$

where ${\varepsilon }_{\mathrm{1,2}}$ are the dielectric permittivities of the contacting media, ${\bf{E}}=-\nabla {\phi }({\bf{r}})$ is the electric field, and indices $n$ and $t$ correspond to the normal and tangential field components, respectively. In our calculations, the dielectric permittivities ${\varepsilon }_{W}=81$ (aqua solvent) and ${\varepsilon }_{S}=4.0$ (glass window) were used^60^.

Solution of Eqs. (2)–(5) can be obtained by using boundary element method^61^. According to Eq. (2), potential ${\phi }({\bf{r}})$ is a harmonic function and, therefore, ${\phi }({\bf{r}})$ satisfies relation^62^

6 $$\phi ({\bf{r}})=\frac{1}{2\pi C}{\int }_{{\rm{\Omega }}}[\frac{1}{|{\bf{r}}-{\bf{r}}^{\prime} |}\frac{{\rm{\partial }}\phi ({\bf{r}}^{\prime} )}{{\rm{\partial }}{\bf{n}}^{\prime} }-\phi ({\bf{r}}^{\prime} )\frac{{\rm{\partial }}}{{\rm{\partial }}{\bf{n}}^{\prime} }(\frac{1}{|{\bf{r}}-{\bf{r}}^{\prime} |})]d{\bf{S}}^{\prime} .$$

Here, the integration is performed over boundary ${\rm{\Omega }}$ (plane $z=0$), $d{{\bf{S}}}^{^{\prime} }$ is a surface element, ${{\bf{n}}}^{^{\prime} }$ is the external normal vector to the surface, ${\rm{\partial }}/{\rm{\partial }}{{\bf{n}}}^{^{\prime} }$ is the derivative along ${{\bf{n}}}^{^{\prime} }$; we use $C=1$ for the potential at boundary ${\rm{\Omega }}$ and $C=2$ for the calculation in the volume.

To find the electric field near the surface of electrodes, the plane $z=0$ is triangulated to small boundary elements. The potential at the boundary is related to the normal component of the electric field in the substrate window, ${{\bf{E}}}_{n}=-{\varepsilon }_{S}^{-1}\partial {\phi }/\partial {\bf{n}}{|}_{{\rm{\Omega }}}$. Assuming that the magnitude of the normal field within a given boundary element is constant, we can apply Eq. (6) to each element. This yields

7 $$\begin{array}{c}{E}_{nj}={G}_{ij}^{-1}{H}_{il}{\phi }_{l},\\ {G}_{ij}=\frac{{\varepsilon }_{S}}{2\pi }{\int }_{{{\rm{\Omega }}}_{j}}\frac{d{S}^{^{\prime} }}{|{{\bf{r}}}_{i}-{{\bf{r}}}^{^{\prime} }|},\quad \quad {H}_{ij}=-{\delta }_{ij}-\frac{1}{2\pi }{\int }_{{{\rm{\Omega }}}_{j}}\frac{{\rm{\partial }}}{{\rm{\partial }}{{\bf{n}}}^{^{\prime} }}(\frac{1}{|{{\bf{r}}}_{i}-{{\bf{r}}}^{^{\prime} }|})d{{\bf{S}}}^{^{\prime} },\end{array}$$

where $i,j,l$ are indices of the boundary elements, ${\delta }_{ij}$ is unit matrix, ${{\bf{r}}}_{i}$ is the center of boundary element $i$, ${{\rm{\Omega }}}_{j}$ is a surface of boundary element $j$, ${G}^{-1}$ is inverse matrix to $G$, and the summation is over repeated indices. Note that the horizontal size of a system should be much larger than thickness $h$ and diameter $D$, to exclude the horizontal boundary effects.

Using Eq. (7) at boundary ${\rm{\Omega }}$, potential ${\phi }({\bf{r}})$ in the volume of the substrate window is readily obtained from Eq. (6) with $C=2$. At $z=h$, Eq. (6) with boundary conditions (5) yield the potential and electric field at the solvent interface. Then one can apply Eq. (6) again, to calculate $\phi ({\bf{r}})$ in the solvent.

Figure 7(b) presents a typical distribution of the electric force lines in the area of interest. The results correspond to $U/D=2\times {10}^{3}V/\text{mm}$. One can see that the electric field is indeed planar in the central area.

### Details of demonstration experiment

All glass surfaces were treated with ethanol and deionized water, and then dried at temperature of $150$ °C during 30 minutes. Afterwards, the optical interfaces were treated to form a hydrophobic coating. The hydrophobization was performed with deposition of 3% solution of polymethylsiloxane oil (PMS-200) in benzol, followed by annealing at 250 °C during 120 minutes.

The colloidal suspension, used for the demonstration experiment shown in Fig. 4, contained silica particles with diameter of $2.12{\mu }{\rm{m}}$ (${{\rm{SiO}}}_{2}$, Microparticles GmbH, Germany), dispersed in deionized water with resistance of $18.2\,{\rm{M}}{\rm{\Omega }}\times {\rm{cm}}$, produced after the purification of distilled water in ion-exchanging resins. The particles were dispersed in the solvent by ultrasonic mixing.
